# Biophysical properties at patch scale shape the metabolism of biofilm landscapes

**DOI:** 10.1038/s41522-022-00269-0

**Published:** 2022-02-03

**Authors:** Anna Depetris, Giorgia Tagliavini, Hannes Peter, Michael Kühl, Markus Holzner, Tom J. Battin

**Affiliations:** 1 River Ecosystems Laboratory, Alpine and Polar Environmental Research Center (ALPOLE), Ecole Polytechnique Fédérale de Lausanne (EPFL), Lausanne, Switzerland; 2 Institute of Environmental Engineering, Department of Civil and Environmental Engineering, ETH Zurich, Zurich, Switzerland; 3 Swiss Federal Institutes for Forest, Snow and Landscape Research (WSL) and Aquatic Sciences and Technology (Eawag), Dübendorf, Switzerland; 4 Marine Biological Section, Department of Biology, University of Copenhagen, Copenhagen, Denmark

**Keywords:** Biofilms, Water microbiology

## Abstract

Phototrophic biofilms form complex spatial patterns in streams and rivers, yet, how community patchiness, structure and function are coupled and contribute to larger-scale metabolism remains unkown. Here, we combined optical coherence tomography with automated O_2_ microprofiling and amplicon sequencing in a flume experiment to show how distinct community patches interact with the hydraulic environment and how this affects the internal distribution of oxygen. We used numerical simulations to derive rates of community photosynthetic activity and respiration at the patch scale and use the obtained parameter to upscale from individual patches to the larger biofilm landscape. Our biofilm landscape approach revealed evidence of parallels in the structure-function coupling between phototrophic biofilms and their streambed habitat.

## Introduction

Biofilms are surface-attached and matrix-enclosed communities that dominate the microbial world in most natural ecosystems^1^ and that are of utmost relevance to public health^2,3^. Owing to their three-dimensional structure, biofilms can be considered as microbial landscapes^4^ and as such are increasingly recognized as an integrated part of the landscape they inhabit^5^. This notion has enabled aquatic microbial ecologists^4^ and more recently also microbiologists studying the human microbiome^6^ to apply landscape ecology theory to the microbial realm. A basic tenet of landscape ecology is that the spatial arrangement of patches reciprocally interacts with higher-order processes such as mass flux, dispersal, and biodiversity dynamics^7,8^. Despite the inherent links between patchiness and the functioning of landscapes, the existence of patches and their functional relevance for biofilms as microbial landscapes remains elusive. This is certainly attributable to the length scales typically used in biofilm research (micrometer to millimeter), which is below the length scale at which patchiness emerges (millimeter to several centimeters)^4,9^. Establishing links between biofilm’s three-dimensional structure, patchiness and higher-order processes is critical to improve our understanding of the success of biofilms^1,5^ and their relevance for ecosystem functioning.

The structure-function coupling of biofilms is modulated to a large extent by the fluid flow around them^10–12^. Whereas flow-induced shear stress can remove biomass locally, flow can increase mass transfer and plays an important role in the establishment of gradients in nutrients as well as electron donors and acceptors and thus the diversification of niches within biofilms^13^. In phototrophic biofilms, such as those that colonize the benthic zone of streams and rivers, these gradients can be reinforced by vertical light gradients^14,15^. Advective transport was shown to enhance solute replenishment via channels that surround individual biofilm clusters^16–18^. Turbulent bursts can also occasionally erode the diffusive boundary layer thereby enhancing solute transport from the bulk liquid to the biofilms^10,19^. Within biofilms, particularly when their permeability is low as in most purely bacterial biofilms, diffusion governs solute transport^10,20,21^. However, in more permeable biofilms, advection can sustain mass transport, provided that the pressure gradient is sufficiently large^22,23^. The enhancement of solute transport in permeable biofilms likely modulates the metabolic activity, however, this has not been investigated for complex phototrophic biofilms.

Phototrophic biofilms in streams harbor diverse communities and develop complex topographies^5,24,25^. They are integrated parts of the hierarchically organized stream ecosystem and its nested levels of spatial heterogeneity^26,27^. These communities are biodiversity hotspots and drive ecosystem metabolism, nutrient, and carbon cycling^5^. While biodiversity dynamics of benthic biofilms are increasingly understood at the scale relevant to typical streambed features (e.g., bedforms, length scales of centimeters to meters)^28,29^, we do currently not understand how microbial diversity is organized at length scales relevant to the structure and functioning of biofilm landscapes (length scales of millimeters to centimeters). This is potentially relevant to infer ecological mechanisms underlying the striking biodiversity with hundreds of microbial taxa coexisting within streambed patches.

This work was motivated by the questions of how physical and biological processes interact to control small-scale patterns of biofilm structure and activity, and how these local patterns influence outcomes at the larger scale, notably at the scale of the biofilm landscape. To address these questions, we grew phototrophic biofilms under two hydraulic environments and combined amplicon sequencing with spatially resolved O_2_ microprofiling guided by optical coherence tomography (OCT). We leveraged the spatial scale of OCT imaging and O_2_ microprofiling using two-dimensional numerical simulations to assess fluid flow and mass transfer. We found that biofilm patches differed in physical structure, mass transfer, as well as community composition, and metabolic activity. Upscaling to the entire biofilm landscape, we estimate the relative contribution of the various patches to community metabolism. Our results indicate how small-scale biodiversity patterns and structure-function coupling affect higher-order biofilm functioning, with possible impacts on stream ecosystem metabolism.

## Results and discussion

### Distinct patch types emerge from the biofilm landscape

**Fig. 1:**
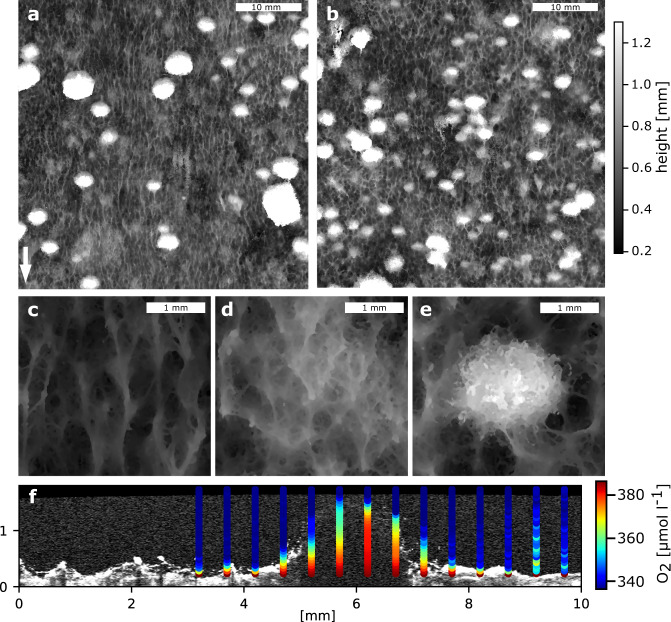
Characterization of a patchy phototrophic biofilm. A phototrophic biofilm was grown from a natural inoculum in an open-channel flume with a turbulent flow regime and a gradient of hydraulic conditions. A digital elevation model of the biofilm surface topology under fast (**a**) and slow (**b**) flow was derived from the OCT dataset. Note the formation of ridges aligned in the flow direction (arrow in panel **a**). High-resolution structural details are shown for an example of the cyanobacteria-dominated base (**c**), a diatom-dominated patch (**c**) and (**d**) Klebsormidiophyceae-dominated patch (**e**). Transects of O_2_ concentration profiles were obtained across selected biofilm structures. An example OCT scan aligned with the corresponding O_2_ concentration measurements over a Klebsormidiophyceae-dominated patch is reported (**f**).

We grew phototrophic biofilms from raw surface water in a flume designed to reproduce a gradient of mean flow velocity and turbulence relevant for the hydraulics of streams (Methods). We focused on two contrasting hydraulic environments: one characterized by high bulk flow velocity (0.13 m s^−1^) and turbulent kinetic energy (7 × 10^−4^ m^2^ s^−2^) and another characterized by low flow velocity (0.06 m s^−1^) and turbulent kinetic energy (2 × 10^−4^ m^2^ s^−2^) (Supplementary Fig. 1). Mature biofilms (after 1 month of in situ growth) consisted of a base layer evenly carpeting the flume bottom, and dominated by cyanobacteria (henceforth referred to as cyanobacteria-dominated basis, CDB) (Fig. 1). Within the biofilm landscape, distinct patches emerged from the CDB, with fluffy tufts (up to 1.5 mm in height) dominated by green algae of the Klebsormidiophyceae family (henceforth referred to as Klebsormidiophyceae-dominated patches, KDP) and interspersed patches dominated by diatoms (henceforth referred to as diatom-dominated patches, DDP).

### Community turnover across patch types

**Fig. 2:**
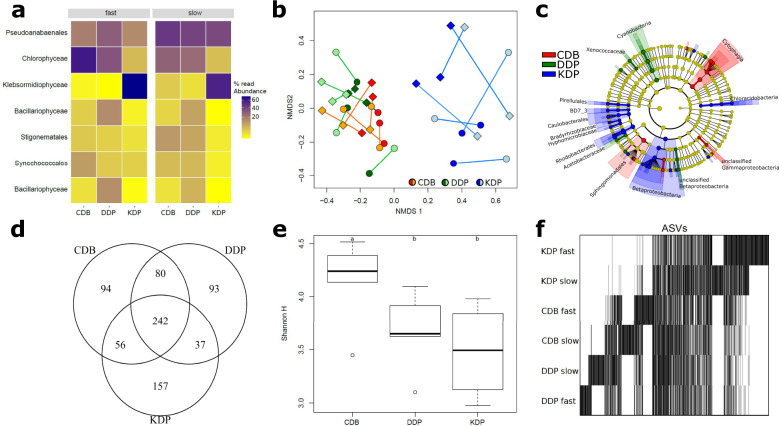
Patch types differ in composition and their contribution to diversity. A heatmap shows the distribution of the most abundant phototrophic community members in CDB, DDP, and KDP (**a**). Non-metric multidimensional scaling with Procrustes superimposition illustrates the compositional similarity of CDB and DDP and the more distinct assemblages in KDP, as well as the coupling between prokaryotic (lighter color) and phototrophic eukaryotic (darker colors) communities grown under fast (diamonds) and slow (circles) flow (**b**). The cladogram depicts taxonomic lineages (prokaryotes) that are significantly enriched in the three patch types (**c**). Note that particularly KDP harbored a distinct set of prokaryotic community members. A Venn diagram shows the number of shared and unique prokaryotic amplicon sequence variants (ASVs) among the three patch types (**d**). While a similar number of ASVs was detected in each patch type, particularly KDP were dominated by abundant prokaryotic ASVs. This is reflected in the significantly reduced Shannon H in KDP (**e**, letters indicate significance in post hoc comparisons). A patch-by-ASV matrix arranged by reciprocal averaging further highlights the turnover and specificity of ASV among patch types (**f**). Prokaryotic ASVs common to all patch types form a central band, whereas ASVs specific to each patch type appear clustered.

Community composition and diversity of stream biofilms have been studied across spatial scales, ranging from entire stream networks^30^ to small-scale streambed localities^28,29^. However, to study biodiversity dynamics relevant to the scale of biofilm landscapes, we individually sampled triplicates of CDB, DDP, and KDP from both hydraulic environments. Using 16 S and 18 S rRNA gene sequencing (Methods), we found a consistent community turnover among all three patch types (Fig. 2a). The CDB communities were dominated by cyanobacteria (predominantly members of the Pseudanabaenales, Synechococcales, and Stigonematales) and a not further classified Chlorophyceae. This unclassified Chlorophyceae even contributed to the majority of 18 S rRNA gene sequences under fast flow, contrary to the microscopic observations and likely attributable to differences in rRNA gene copy numbers of cyanobacteria and the unclassified Chlorophyceae. The DDP community was dominated by the pennate diatom *Achnanthidium saprophilum*, and the KDP community by a not further classified filamentous algae of the Klebsormidiophyceae clade.

Besides these major phototrophs that did not differ between both flow environments, CDB, DDP, and KDP were each associated with diverse eukaryotic and bacterial communities. For instance, between 36 and 46 distinct eukaryotic community members were detected in the CDB, KDP, and DDP, respectively. Bacterial heterotrophs were more diverse, with 140 to 281 amplicon sequence variants (ASVs) present in the three patch types. Generally, the bacterial assemblages were specific to CDB, DDP, and KDP, with Bray–Curtis similarities among the three replicates of the same patch type averaging 0.62 ± 0.10, while similarity across different patch types averaged 0.48 ± 0.11 (ANOSIM, *R* = 0.63, *p* = 0.01). Differences in abundance-based community similarity across samples can arise from the replacement of individuals of some taxa by individuals of other taxa (i.e., balanced variation in abundance) or through abundance gradients, in which abundance changes between samples without taxa substitution. Partitioning Bray–Curtis similarities into these two components, we found that balanced variation in abundance dominated among replicate-dissimilarity, accounting on average for 59.3 ± 10.8 and 62.1 ± 9.1% of community turnover in CDB and DDP, whereas this process accounted on average for 91.4 ± 7.4% of the differences observed among replicates in KDP. Differences in Bray–Curtis similarity across patch types were also predominantly (82.0 ± 26.6%) attributed to balanced variation in abundance. Taken together, these results highlight that all three patch types harbor distinct and specific communities with the substitution of taxa being the dominant driver of compositional turnover within the biofilm landscape.

Non-metric multidimensional scaling (NMDS) ordination combined with Procrustes superimposition revealed an overlap between the CDB and DDP community compositions, while both differed from the KDP community (Fig. 2b). This analysis also highlighted the significant coupling between eukaryotic and bacterial communities in each patch type (correlation in a symmetric Procrustes rotation: 0.69, *p* < 0.01). Furthermore, using linear discriminant analysis effect sizes (LefSe)^31^ (Fig. 2c), we identified taxonomic units that were specifically enriched in the three patch types. Several cyanobacterial taxa were enriched in the DDP, and members of Cytophagia, unclassified Gammaproteobacteria, and Sphingomonadales were enriched in CDB (Fig. 2c). However, KDP had the largest number of consistently enriched taxa, particularly affiliated with Betaproteobacteria (Burkholderiales) and Alphaproteobacteria (Caulobacterales, Rhodobacterales, Bradyrhizobiaceae, and Hyphomicrobiaceae).

The specificity of the bacterial community was also reflected in terms of diversity (Fig. 2d). While 31.8% of all ASVs were found in all patch types, between 12.3 and 20.7% of ASVs (accounting in total for 45.3% of ASVs) were exclusively detected in either CDB, DDP, or KDP (Fig. 2d). There were no significant differences in terms of ASV richness between the three biofilm components under the two flow regimes, yet, bacterial community diversity incorporating evenness (Shannon H) was significantly lower in KDP and DDP as compared to CDB (ANOVA, *F* = 11.78, *p* < 0.01) (Fig. 2e), reflecting the relative dominance of a few bacterial ASVs in these patches. Generally, we found small differences in community composition and diversity between the two hydraulic regimes. These results show that diversity across the entire biofilm landscape was composed of a core of abundant and common ASVs, but that nearly half of the ASV diversity was exclusively found in the different patches.

Collectively, these results highlight the nested contribution of patch types to overall biofilm diversity. This is analogous to real landscapes and stream ecosystems, where biodiversity patterns across scales (e.g., from the stream network to the streambed patch) arise from local communities that assemble from a regional species pool^28,30^. Environmental variation and interactions between hydraulic and microbial processes have been evoked to induce heterogeneity and patchiness of the streambed landscape^27,32^ and its biofilms^5^. Strikingly, however, in our experiments, patchiness arose in the absence of substrate heterogeneity, which highlights the importance of auto-generated structural differentiation of biofilms. Auto-generated differentiation, such as the formation of ridges, can result from the coupling of localized growth, flow conditions, and mass transfer^2^, which may arise stochastically (e.g., through priority effects) or through the interactions between neighboring microcolonies even early in biofilm formation^33,34^. While initial patchiness may be the result of stochastic processes, patches are likely stabilized by shared metabolites among mutualistically interacting taxa or inhibitory modification of niches^35^. In the context of phototrophic stream biofilms, this highlights the importance of interactions among phototrophs and heterotrophs as potential drivers of patchy biodiversity patterns.

### Biofilm patches have distinct physical structures that interact with fluid flow

To explore the physical structure of the three patch types, we inferred thickness, coverage, and volume from OCT imaging^36^ (Methods). We did not find significant effects of the hydraulics on the overall thickness, areal coverage, and volume of the biofilm, neither at the landscape level nor at the level of individual patch types (Table 1). Likely, the differences in the imposed hydraulics did not suffice to substantially compress the biofilms nor to select for a flow-resistant biofilm under fast flow. However, more subtle differences among hydraulic conditions could be noticed. For instance, under high flow velocity, CDB tended to develop into ridges aligned in the flow direction, as was previously reported for similar phototrophic exposed to unidirectional flow^12,33^, while KDPs were fewer but taller and larger in volume (Fig. 1 and Table 1).

**Table 1:** Biofilm features across patch types and flow conditions, as derived from OCT and macro-photography imaging. A total area of 2016 mm^2^ under slow and 1601 mm^2^ under fast flow was analyzed.

Flow	Patch type	Height (mean ± SD) [mm]	Area [mm²]	Coverage [%]	Volume [mm³]	Volume [%]
Slow	CDB	0.39 ± 0.15	1411	70.0	591	57
	DDP	0.58 ± 0.15	408	20.2	253	25
	KDP	1.09 ± 0.24	197	9.8	189	18
Fast	CDB	0.38 ± 0.11	1145	71.5	440	57
	DDP	0.51 ± 0.13	314	19.6	174	23
	KDP	1.27 ± 0.27	142	8.9	152	20

The internal porosity of biofilms is relevant for mass transfer, chemical heterogeneity, and hence for the functioning of biofilms^2^. However, it is notoriously difficult to empirically estimate the porosity within biofilms^36,37^. Using OCT scans (Methods), we estimated the apparent porosity of all three patch types ranging from 0.29 ± 0.22 to 0.71 ± 0.15 in CDB and KDP, respectively (Supplementary Table 1). We found that KDP porosity was ~1.5 times greater than CDB and DDP porosity, which is in line with the filamentous and hence porous structure of this patch type as revealed by Confocal Laser Scanning Microscopy. Our analyses did not reveal the effect of hydraulics on biofilm porosity.

**Fig. 3:**
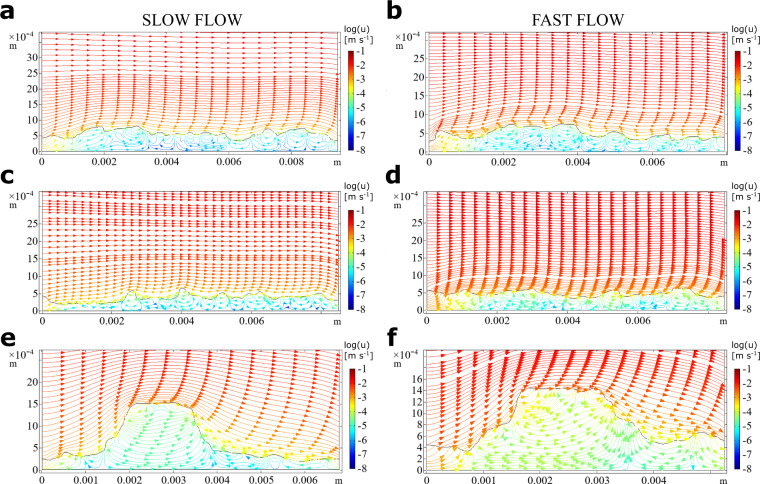
Fluid motion within and around biofilms. We used numerical simulations to compare the fluid patterns within and around biofilm patch types. Streamlines plots of the velocity magnitude (log10(u)) are shown for slow (left) and fast flow (right) conditions for DDP (**a**, **b**), CDB (**c**, **d**), and KDP (**e**, **f**). Note that streamlines in the water domain closely track the biofilm topology but are affected by tall, protruding structures. Significant flow patterns within the biofilm domain emerged at the scale of the biofilm structural features and affected transport within the biofilms.

Biofilms not only respond to but also influence the adjacent flow environment^17,38,39^. To assess how the different patch types interact with the fluid dynamics, we numerically simulated the flow field within and around each of them (Methods). We stress that these simulations were performed on two-dimensional biofilm topologies and hence do not resolve three-dimensional flow around patches. We acknowledge that the two-dimensional model is a simplification of reality, but it captures the main physics and qualitative features of the system. We found that the three patch types differently deflected streamlines, both up- and downstream and under both flow environments (Fig. 3). This effect was particularly evident for the protruding KDP, where streamlines also detached to develop a downstream wake.

This interaction between the local biofilm topography and flow affected the distribution of the viscous sublayer thickness. Our simulations estimated the thickness of the viscous sublayer at 0.13 and 0.07 mm under slow and fast flow, respectively. It was generally thinner at the top of KDP (~0.04 mm), than both upstream (0.10 mm) and downstream (0.14 mm) of this patch type.

Our simulations further revealed significant fluid flow within the biofilm (Fig. 3). Flow patterns were consistently characterized by liquid entering the microcolonies upstream and exiting them downstream. For instance, in both hydraulic environments, the fluid entered the KDP and traversed it almost parallel to the bulk flow lines, which, albeit also affected by biofilm physical structure, seems to reflect high permeability. Numerically estimated permeability varied between 0.5 × 10^−10^ m^2^ and 2 × 10^−10^ m^2^, and did not differ between KDP and the other two patch types. The internal fluid velocity was highest in KDP (0.046 mm s^−1^), followed by CDB (0.041 mm s^−1^) and DDP (0.031 mm s^−1^) under slow flow. Under fast flow, internal fluid velocity increased to 0.170, 0.137, and 0.061 mm s^−1^, for KDP, CDB, and DDP, respectively. We attribute these differences to the tall and protruding structure of KDPs, which are impacted by a higher velocity upstream (high pressure) and induce a wake downstream (low pressure) that results in a streamwise pressure gradient. These estimates of internal flow velocity and permeability are higher than those reported from mono-species bacterial biofilms^11^. We attribute this difference to the abundance of algal cells, which owing to their large size and often filamentous structure increase the porosity of phototrophic biofilms. These findings highlight the importance of advection for mass transport within phototrophic biofilms. Previous studies demonstrated the importance of advective transport of solutes from the bulk liquid to the surface of, for instance, algal mats^40^, while diffusion dominated internal transport. Our spatially highly resolved observations and simulations highlight the role of advective transport within microcolonies protruding into the bulk liquid. Our findings specifically reveal advection to supply oxygen to deeper biofilm layers and to evacuate oxygen-depleted water from the microcolonies on their downstream side.

These findings on internal flow patterns are reminiscent of the hydrodynamic exchange of mass and solutes across streambeds, particularly their bedforms, and present evidence towards the congruence of the structure-function coupling between biofilms and their habitat^5^. While differing in spatial scale, both, biofilm microcolonies and bedforms, are porous systems where hydrodynamics drives advective flow, which enhances mass transport and microbial activity^41,42^. Although pressure differences underly flow in both bedforms and biofilms, pressure differences across geomorphological features (e.g., step-pools in mountain streams) typically arise from differences in water height (i.e., static pressure), whereas in the case of biofilm patches, internal advection arises because of dynamic pressure. We propose that similar structure-function coupling across spatial scales contributes to the high retention and transformation capacity of stream ecosystems—a notion that is in line with the “benthic biolayer” just below the sediment-surface interface in streams where reaction rates are highest, and that greatly contributes to solute dynamics at the scale of the entire streams^43^.

### Patch type and hydraulics affect O_2_ dynamics

**Fig. 4:**
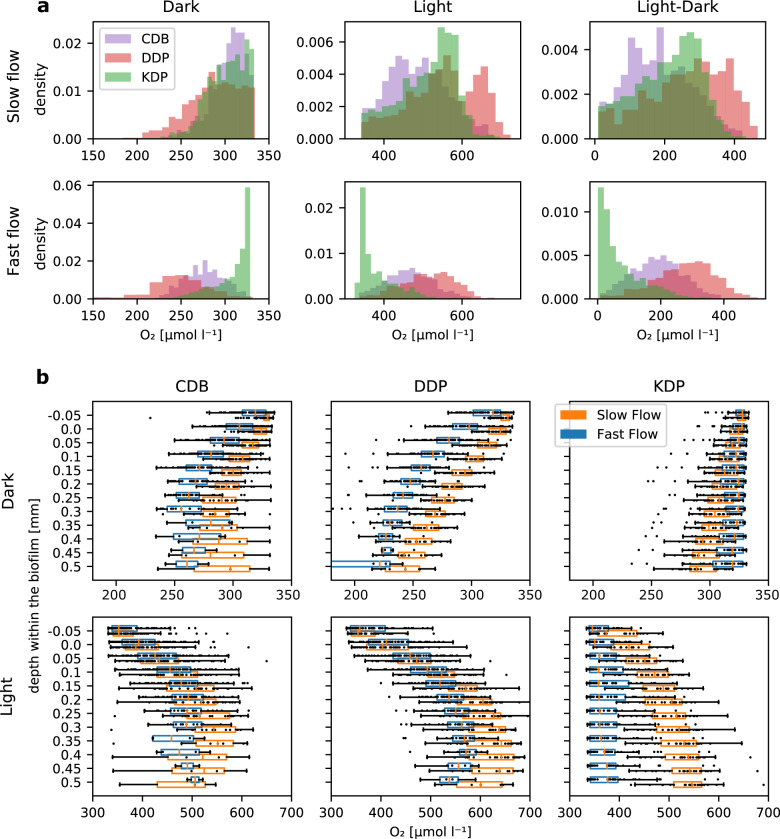
O_2_ dynamics at the landscape scale. O_2_ concentration obtained by microelectrode profiling revealed the impacts of hydraulic conditions and patch type under light and darkness (and the variation between light and dark conditions). Pooled probability density distributions of O_2_ concentration measurements are shown (**a**). Flow, light/dark conditions, and patch type also affected the vertical distribution of O_2_ within biofilms (**b**). Note the differences among y-axis scales and the divergence of the impact of the hydraulic condition on the vertical distribution towards greater depth within the biofilm.

The spatiotemporal dynamics of O_2_ concentration in biofilms result from mass transport (i.e., replenishment) and microbial activity (i.e., consumption and production)^10,11^. To assess O_2_ dynamics at the high spatial resolution, we used OCT-guided, automated microelectrodes profiling and obtained a total of 1134 depth profiles of O_2_ across all three patch types, both in light and dark conditions, as well as in both hydraulic environments (Methods). We found that the distribution of O_2_ concentration within the biofilm, and its variation between light and dark conditions, differed among patch types (Wilcoxon rank test, *p* < 0.01 for each comparison) (Fig. 4a); with the exception of KDP compared to CDB under dark conditions (Wilcoxon rank text, *p* = 0.17) (Fig. 4a). Under both flow conditions, DDP exhibited the largest ranges in O_2_ concentration and the greatest light-to-dark differences.

Given the importance of advection, both in- and outside the biofilm, we posit that high flow velocity should reduce the magnitude and dampen the variation of oxygen gradients within biofilms. Indeed, in all three patch types, and under both light and dark conditions, faster flow velocities significantly decreased variation in O_2_ concentration (Wilcoxon rank test, *p* < 0.01, Fig. 4a), with the exception of KDP in the dark. In the most permeable biofilm patch type, KDP, the distribution of O_2_ concentration was narrow and skewed towards the bulk water O_2_ concentration. Similarly, light-to-dark differences in KDP were also diminished under fast flow compared to slow flow. Under fast flow, CDB and DDP reached lower O_2_ concentrations in the dark throughout the entire biofilm thickness compared to slow flow (Fig. 4b). In contrast, biofilm grown under slow flow accumulated more O_2_ in deeper layers (under light). Moreover, we observed that O_2_ concentration consistently peaked ~0.35 mm into the DDP patches, and decreased deeper into the biofilm. Overall, the most permeable biofilm patch type, KDP, presented weaker O_2_ gradients whereas CDB and DDP presented a strong vertical differentiation of the chemical microenvironment.

### Patch type affects O_2_ transport

**Fig. 5:**
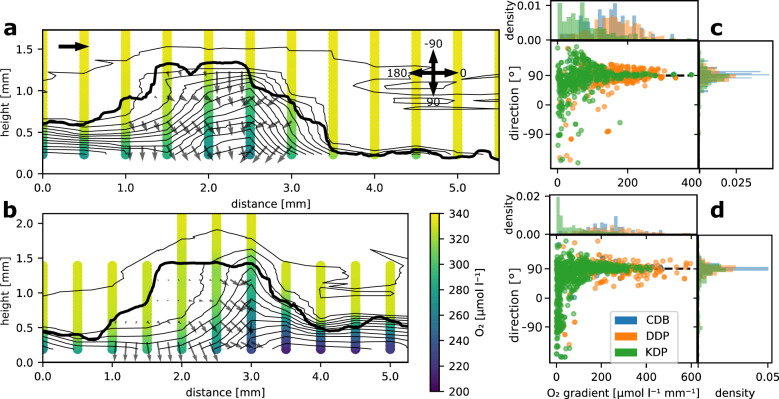
O_2_ dynamics at the patch scale. We measured several transects of O_2_ concentration profiles across the three patch types. Examples of profile transects measured over KDP (in darkness) under slow (**a**) and fast (**b**) flow are shown. The vertical profiles indicate the measured O_2_ concentration, contour lines show the O_2_ concentration field triangulated from these measurements, while the gray arrows indicate the intensity and direction of the O_2_ concentration gradient. The top-left arrow indicates the direction of the bulk fluid flow. The direction of the O_2_ concentration gradients (in darkness) under slow flow (**c**) and fast (**d**) flow were compared within several replicates per patch type. See arrow cross in panel **a** for directions. While CDB and DDP gradients were steep and directed towards the substrate (90°, black dashed line), O_2_ gradients within KDP were less intense and often bent against the flow direction (>90°).

In order to explore patterns in O_2_ transport within and above biofilm patches, we established transects of O_2_ concentration profiles across the different patch types, aligned with the flow direction (Methods). We found that O_2_ concentration above the biofilm was relatively homogeneous, which indicates that the diffusive boundary layer was thin and followed the biofilm surface topology (Fig. 5a).

Furthermore, we found that flow had a remarkable effect on the distribution of measured O_2_ concentrations within KDP. We observed an elevated O_2_ concentration upstream and reduced O_2_ concentration downstream within patches (in the dark); this effect was exacerbated under fast flow (Fig. 5b). This pattern supports the finding that notable advective transport drove O_2_ dynamics within KDP. In contrast, O_2_ gradients in CDB and DDP were overall more directed towards the substrate, and hence perpendicular to their main flow direction (Fig. 5c, d).

**Fig. 6:**
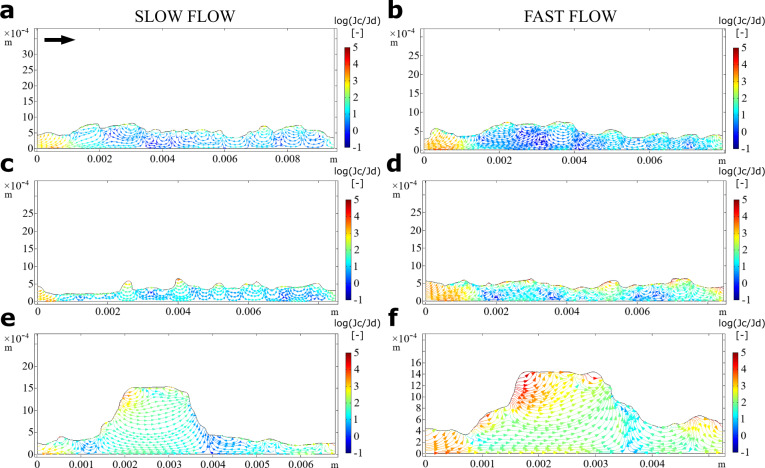
O_2_ transport within biofilms. We used numerical simulations to compare the mass transport patterns within and around the biofilm patch types developed under contrasting hydraulic conditions. Streamline plots for slow and fast flow conditions for DDP (**a**, **b**), CDB (**c**, **d**), and KDP (**e**, **f**) are shown. The streamline plots depict log10 of the ratio between advective (Jc) and diffusive (Jd) fluxes within the biofilm.

To gain a better mechanistic understanding of the patterns of O_2_ concentration observed within patches, we numerically simulated O_2_ production in the light (i.e., net photosynthesis, NP) and consumption in the dark (i.e., respiration, R), and transport within and around the various patch types (Methods). The parameters in these simulations were optimized such that the modeled O_2_ concentration profiles best predicted observed O_2_ profiles (Table 2). We found that the modeled diffusive boundary layer thickness averaged 6 and 3 μm under slow and fast flow, respectively, and overall followed the patterns of the above-mentioned viscous boundary layer. This is in line with our microsensor measurements (Supplementary Figs. 2, 3), and is explained by turbulent mixing (estimated eddy diffusivity reached ~10^−5^ m^2^ s^−1^ for both flow environments). In agreement with the simulated patterns of fluid flow, we found that advective transport greatly exceeded diffusive transport of O_2_ in the biofilms (Fig. 6), which was particularly evident in KDP, where the average ratio between advective and diffusive fluxes reached 86.7, compared to 76.7 and 53.8 in CDB and DDP, respectively. This was further exacerbated under high flow velocity, where the average ratio between advective and diffusive transport reached 275.8, 141.7, and 62.1 for KDP, CDB, and DDP, respectively. Furthermore, the fluid flow patterns may have greatly enhanced solute exchange between the biofilm and the bulk fluid. Accordingly, we found that advection accounted for 98.9% of the total O_2_ flux across the biofilm surface in KDP, for 98.2 and for 98.7% of the total flux in DDP and CDB, respectively. This fraction increased under fast flow velocity (KDP: 99.6%; DDP: 98.4%; CDB: 99.3%). Overall, our results highlight that advection enhanced O_2_ transport into and out of all three patch types.

**Table 2:** Optimized model parameters that best predicted the measured transects of O_2_ concentration profiles across different patch types and flow conditions.

Flow	Patch type	Porosity	O_2_ diffusivity (D_eff_/D_aq_)	Permeability [m^2^]	Turbulent Prandtl number	Eddy diffusivity [m^2^ s^−1^]
Slow	CDB	0.36	0.7	1 × 10^−10^	0.98	1 × 10^−5^
	DDP	0.47	0.7	0.6 × 10^−10^	0.6	1.2 × 10^−5^
	KDP	0.58	0.8	1 × 10^−10^	0.95	1.4 × 10^−5^
Fast	CDB	0.62	0.7	1 × 10^−10^	1	1.8 × 10^−5^
	DDP	0.41	0.5	0.5 × 10^−10^	1	1.2 × 10^−5^
	KDP	0.86	0.9	2 × 10^−10^	0.72	1.2 × 10^−5^

### Metabolism from the patch scale to the biofilm landscape

Extrapolating fluxes from the patch to the landscape scale is critical in order to understand the relevance of the various landscape constituents to overall functioning^44^. To achieve this, we combined numerically simulated volumetric rates of net O_2_ production (NP) and respiration (R) with OCT-derived estimates of the volume and areal coverage of each biofilm patch type (Methods). Among all patch types and flow conditions, volumetric NP ranged from 1.1 to 5.38 mmol O_2_ m^−3^ s^−1^ and R ranged from −0.55 to −1.61 mmol O_2_ m^−3^ s^−1^. R was generally higher under fast compared to slow flow (Table 3). Integrating these fluxes over the entire biofilm landscape, we estimated that in the dark, the biofilm consumed on average 0.69 mmol O_2_ m^−2^ s^−1^ and 0.44 mmol O_2_ m^−2^ s^−1^ under fast and slow flow, respectively. On the other hand, the average O_2_ production under light (i.e., NP) was 1.8 mmol O_2_ m^−2^ s^−1^ and 2.11 mmol O_2_ m^−2^ s^−1^, under fast and slow flow, respectively. These volumetric rates and areal fluxes are closely bracketed by those computed from microsensor profiles measured in microbial mats formed by cyanobacteria and diatoms in alluvial rivers^40^ and salt marshes^45^ and by epibenthic biofilms^14,46^. We consider this as a confirmation that our approach of microelectrode measurements and numerical modeling provided reliable estimates of metabolic fluxes. Furthermore, we found that the contributions from CDB, DDP and KDP to the landscape-level metabolism largely reflected their relative contribution by volume (Table 3). However, it is clear that besides areal coverage, small-scale spatial covariation of microbial activity and flow in both benthic and hyporheic zones of streams and river influences the bioactive layer extent, with important consequences for reach-scale biogeochemical transformations^41,43,47^.

**Table 3:** Modeled volumetric rates [mmol m^−3^ s^−1^] of O_2_ consumption in the dark (R) and net O_2_ production in the light (NP) were used to estimate the net O_2_ balance (NP–R) of each patch type. O_2_ fluxes (across the projected, planar area) [mmol m^−2^ s^−1^], were calculated based on the aerial coverage and volume of each patch type (Methods). These values were used to estimate the overall O_2_ balance of the whole microbial landscape, both as volumetric rate (vol.) and aerial flux (area).

Flow	Patch type	R (vol.) [mmol m^−3^ s^−1^]	NP (vol.) [mmol m^−3^ s^−1^]	NP–R (vol.) [mmol m^−3^ s^−1^]	R (area) [mmol m^−2^ s^−1^]	NP (area) [mmol m^−2^ s^−1^]	NP–R (area) [mmol m^−2^ s^−1^]
Slow	CDB	−0.98	5.1	4.11	−0.41	2.13	1.72
	DDP	−0.55	3.0	2.46	−0.34	1.87	1.53
	KDP	−0.88	2.5	1.66	−0.84	2.42	1.58
	landscape	−0.86	4.11	3.26	−0.44	2.11	1.67
Fast	CDB	−1.61	5.38	3.77	−0.62	2.06	1.45
	DDP	−1.11	2.06	0.94	−0.62	1.14	0.52
	KDP	−1.37	1.1	−0.28	−1.48	1.18	−0.3
	landscape	−1.45	3.77	2.32	−0.69	1.8	1.11

We next calculated the net O_2_ balance as the difference between R and NP of the biofilm patch types. We found that all three patch types were net producers of O_2_, with the notable exception of KDP under fast flow (Table 3). The positive O_2_ balance is in general agreement with previous findings on cyanobacterial biofilms from hypersaline mats^48,49^, for instance. In streams, ecosystem metabolism is generally net heterotrophic except for certain windows, such as in spring, when copious algal growth can shift ecosystem metabolism to autotrophy^50^. We argue that our benthic biofilms with their marked positive O_2_ balance are representative of the phototrophic biofilms during these windows—potentially even overwhelming the heterotrophy within the deeper layers of streambeds.

Despite limited community turnover and similar biofilm structures across flow regimes, the hydraulic conditions strongly affected the metabolic rates. While R was higher in all three patch types under fast flow than under slow flow, NP was lower under fast flow in two out of the three patch types. The opposite response of R and NP to the hydraulic environment indicates different factors controlling photosynthesis and respiration in phototrophic biofilms. We attribute this to enhanced advective flow that replenishes resources, which otherwise would limit respiration^41,47^. At the landscape scale, NEP under fast flow was 66% lower than under slow flow, an effect that was linked both to a decreased NP and enhanced R. Hence, our results indicate that higher flow velocity, potentially mediated by the delivery of nutrients to biofilm communities, can trigger a shift towards a less net autotrophic metabolism and that these patch-level processes may integrate to a landscape-level metabolic response.

## Conclusion

Combining OCT imaging with microelectrode profiling, numerical simulations, and amplicon sequencing, we highlight the structural and functional patchiness of a phototrophic biofilm. While patchiness in streams is often related to a heterogeneous environment^26,27^, our findings suggest that small-scale patchiness can also exist in a relatively homogeneous environment, potentially emanating from biotic interactions or stochastic processes. While the spatial patchiness of physical structures in mono-species biofilms has been acknowledged for decades, it has been rarely addressed for complex phototrophic biofilms harboring hundreds if not thousands of microbial taxa. We found that phototrophic biofilms emerge as complex landscapes, in which different communities coexist within spatially segregated patches. The functioning of these patches is linked to their structural features, which, by interacting with fluid flow, generate physicochemical micro-niches within the biofilm landscape. Our findings highlight the role of hydraulics, that allows for pervasive advective transport around and within biofilm patches, and that can induce a metabolic shift at the scale of the entire biofilm landscape. Our landscape approach has revealed patchiness as a hitherto unrecognized driver of diversity in natural phototrophic biofilms and the interactions of patches with the fluid dynamics as a driver of their metabolism. The notion of biofilms as microbial landscapes^4–6^ is critical to predict and understand their function and role in ecosystem processes. In this context, our findings shed new light on the biofilm machinery of stream ecosystem metabolism and its consequences for large-scale biogeochemical fluxes.

## Methods

### Biofilm cultivation

We grew a benthic biofilm in an open-channel flume fed by filtered water from Lake Geneva (nominal pore size 50 μm, FA 10 SX 50 ATLAS FILTRI)^36^. A constant flow rate (2.22 × 10^−4^ m^3^ s^−1^) was supplied using valves equipped with flow meters. Water was partially recirculated in a 1 m^3^ reservoir and water temperature varied between 14.5 and 15.5 °C throughout the experiment. Total dissolved organic carbon (DOC) averaged 824 ± 65 ppb and was quantified in filtered samples (pre-ashed GF/F, Whatman) using a TOC carbon analyzer (Sievers M9 TOC Analyser, GE). Phosphate, ammonium, nitrite, and nitrate were measured with a Lachat QuikChem 8500 flow injection analyser (QuikChem Methods 10-115-01-1-M (PO_4_^3−^), 10-107-04-1-B (NO_3_^−^/NO_2_^−^), 10-107-05-1-C (NO_2_^−^) and 10-107-06-3-D (NH_3_)). Average concentrations were 3.75 ± 0.75 ppb, 36 ± 11 ppb, 386 ± 22 ppb for PO_4_^3−^, NH_4_-N, and NO_3_-N, respectively. Hence, C:N, C:P, and N:P ratios were 2.3, 567, and 249, respectively. Light (~13 W m^2^, as measured with a JAZ spectrometer Ocean Optics) was provided for 12 h a day using a combination of red and blue LEDs.

The flume was constructed from plexiglass with a funnel-like shape, with flume width gradually increasing from 0.05 to 0.3 m in the flow direction, resulting in a gradient of decreasing flow velocity. It is important to note that hydraulic conditions at the sites where samples were taken were therefore not independent from another. The average water depth was 0.022 m. The mean flow velocity (u) was estimated from the flume geometry (w), the water depth (h), and discharge (Q, 2.22 10^−4^ m^3^ s^−1^) as in Eq. (1).

1 $$u = \frac{Q}{{h \times w}}$$

and decreased from 0.13 to 0.06 m s^−1^. Flow velocity depth profiles and root-mean-square velocity fluctuations at the extremes of the hydraulic gradients were measured by Laser-Doppler velocimetry in a clean flume without biofilm, which enabled calculation of flow velocity and turbulent kinetic energy (averaged between 0 and 10 mm from the plexiglass surface). Turbulent kinetic energy (TKE) was calculated as in Eq. (2).

2 $${{{\mathrm{TKE}}}} = \frac{1}{2}(u{^{\prime {2}}} + v{^{\prime {2}}} + w{^{\prime {2}}} )$$

Where u′, v′, and w′ denote root means square (RMS) of velocity components. Strong turbulent eddies characterized the flow patterns, which were considerably unsteady.

Prior to the experiment, phototrophic biofilms growing continuously in the reservoir were harvested and disaggregated by shaking. The slurry was filtered (41-μm nylon filter, Millipore) and diluted in 8 L lake water. The biofilm slurry was then poured into the flume and incubated for 12 h without flow (under light). This seeding step produced a thin layer of base biofilm evenly covering the flume bottom. After seeding, the flow was started and biofilm was allowed to grow without disturbance for 30 days.

### OCT data and macro-photography acquisition and processing

After 30 days of biofilm growth, we used a spectral-domain optical coherence tomography system (GANYMEDE, Thorlabs GmbH, Germany) centered at 930 nm (LSM03 lens) and equipped with an immersion adapter. In OCT imaging, an A-scan describes a single vertical profile of laser light interference, whereas a B-scan refers to the combination of several A-scans into a cross-sectional tomographic image. The 3D OCT datasets were acquired by averaging 3 A-scans with an x-y-z resolution of 11 μm × 11 μm × 2.18 μm, covering a scan volume of 10 mm × 10 mm × 2.23 mm. The OCT scans resolved the highly reflective Plexiglas surface but did not completely resolve biofilm structures exceeding 2.23 mm height. We mounted the OCT probe on a precision positioning device (STEPCRAFT, 30 μm precision), as described previously^36^. Automatic positioning and OCT scan acquisition allowed us to obtain 6 × 6 OCT scans in a mosaic pattern (with a 30% overlap of the field of view) at both ends of the velocity gradient. Individual OCT datasets were processed to obtain a digital elevation model (DEM) of the biofilm surface topology that was denoised with a median filter (size of 4 pixels) and stitched^36^. The final DEMs each covered a field of ~ 45 mm × 45 mm. Biovolume was estimated as the volume beneath the biofilm surface, (i.e., the sum of all pixels in the DEM). This was supported by visual inspection of the OCT images, which did not reveal the presence of large void areas (i.e., channels) below the biofilm surface. Median biofilm thickness and inter-quantile range (calculated between the 0.2 and the 0.8 quantiles) were calculated from the distribution of pixel gray-values in the DEM.

Similarly, we mounted a camera (Canon EOS 7D Mark II) equipped with a macro-objective on the precision positioning robot and acquired a large set of pictures in a mosaic pattern, which was assembled using the software Image Composite Editor Version 2.0.3.0 (Microsoft Corporation). We used this picture to segment the three patch types (CDB, DDP, and KDP) based on coloration. More specifically, CDB covered most of the flume, while DDP were identified imposing a threshold on the ratio between the red and green channels. Then, KDP were identified by thresholding a combination of the original green channel and a local mean filter on the same channel. Then, small objects and holes were discarded and the final KDP binary mask was dilated by 5 pixels. The remaining areas were labeled as CDB. The outcome of this segmentation algorithm was confirmed by visual comparison and further substantiated by rRNA gene sequencing. Macrophotographs and OCT-derived DEMs were aligned manually, which allowed us to estimate the thickness distribution of each patch type. An example of the output of this segmentation algorithm is shown in Supplementary Fig. 4.

### Porosity estimate from OCT scans

The porosity of the three patch types was estimated from the OCT scans as the ratio of small void spaces and biomass. To differentiate between areas with and without biomass, we segmented the OCT scans based on two gray-level thresholds (i.e., 120, 130). Gray-level values above the threshold were treated as biomass, whereas gray-level values below were regarded as empty space within the biofilm. The choice of a threshold is somewhat arbitrary and we selected these two values to quantify this uncertainty (both values are well apart from the low gray-level noise outside the biofilm and higher gray-levels deep in the biofilm). OCT signal intensity gradually decreases inside biofilms and therefore, we restricted our analysis to the top layer (two thicknesses, 0.1 and 0.2 mm) and excluded structures protruding beyond the imaging depth of the OCT system (~1.2 mm). Porosity was then estimated as the ratio between biomass volume and the total volume in each position in the OCT scan (Supplementary Table 1 and Supplementary Fig. 5), and the median porosity was calculated within each patch type.

### Microsensor measurement and O_2_ concentration profiles analysis

Vertical depth profiles of O_2_ concentration were measured with a fast-responding Clark-type O_2_ microsensor (tip diameter 50 μm, OX-50, Unisense A/S, Aarhus, Denmark)^51^ in steps of 50 μm. The microsensor was linearly calibrated from sensor readings in air-saturated water and anoxic sodium ascorbate solution. Sensor drift during the experiment was compensated by linear interpolation of the bulk water oxygen concentrations. We noted a gradual increase in bulk water O_2_ concentration during the measurements under light conditions, which we attribute to photosynthetic activity in the flume and header tank. This was reversed after switching to dark conditions but may have led to a slight overestimation of bulk water O_2_ concentrations as compared to theoretical expectations. The microsensor was vertically mounted on a motorized micromanipulator (Unisense A/S) and connected to a laptop-interfaced microsensor multimeter (Unisense A/S). Data acquisition and micromanipulator positioning were controlled by dedicated software (Sensor TracePro, Unisense A/S). The micromanipulator was mounted on the precision positioning robot alongside the OCT probe. In order to calibrate the position of the microsensor with respect to the OCT scans, a parafilm membrane was fixed at 2 mm from the plexiglass surface, and four holes were pierced in it using the microsensor tip. The holes in the membrane were then scanned by OCT in order to calculate the relative shift in x and y directions of the microsensor relative to the OCT scan. Profiles were measured in both fast (*n* = 301) and slow (*n* = 293) flow conditions, following an experimental design including both points- and transects- measurements (Supplementary Fig. 6). Profiles were measured in light and dark conditions, allowing for the O_2_ gradients to stabilize for at least one hour between light conditions. Supplementary Figs. 2 and 3 report two examples of transects of O_2_ concentration profiles aligned with the OCT B-scans and macrophotographs. The position of the biofilm surface was recorded in a few microsensor profiles using an endoscope. This estimate was further refined based on the inflection point of profiles in dark and light conditions, as aligned with the OCT B-scans (Supplementary Fig. 6).

To test for differences in the distributions of O_2_ concentration measured inside each patch type (*n*_CDB fast_ = 131, *n*_CDB slow_ = 141, *n*_DDP fast_ = 76, *n*_DDP slow_ = 78, *n*_KDP fast_ = 68, *n*_KDP slow_ = 49), we pooled all the measurements below the biofilm surface by patch type, light/dark condition and flow condition. We compared distributions among different patch types within the same flow and light conditions using the Wilcoxon rank test. Using the same statistical test, we compared distributions between flow conditions, within the same patch type and light condition.

To test for statistical differences in the distributions of O_2_ concentration at the same depth below the biofilm surface, we aligned each profile based on the latter. Then, we compared measurements at the same depth below (or above) the biofilm surface using unpaired Welch two-sample *t*-tests. To analyze within-patch O_2_ dynamics, we triangulated measurements within transects of O_2_ concentration profiles in each patch type and flow condition (in the dark) and measured the intensity and direction of the two-dimensional O_2_ concentration gradients for each measurement point.

### DNA extraction, sequencing, and bioinformatics

We sampled biofilms exposed to two contrasting hydraulic conditions using sterile razor blades and dissected biofilms into patch types under a microscope (samples were ~5 mm × 5 mm). Three replicates of each patch type and under both flow conditions (*n* = 18) were flash frozen at −80 °C and the DNA was isolated using the DNeasy Power Soil kit (QIAGEN). The 16 S and 18 S rRNA genes were amplified using PCR with the 341 f (5′CCTACGGGNGGCWGCAG-3′) and 785r (5′-GACTACHVGGGTATCTAAKCC-3′)^52^ and TAReuk454FWD1 (5′-TCGTCGGCAGCGTCAGATGTGTATAAGAGACAG-3′) and TAReukREV3 (5′-GTCTCGTGGGCTCGGAGATGTGTATAAGAGACA-3′)^53^ primer pairs, for prokaryotic and eukaryotic community members, respectively. We prepared sequencing libraries using the Nextera XT kit (Illumina), equimolar pooled and sequenced on a 300 bp paired-end MiSeq (Illumina) run at the Lausanne Genomic Technology Facility (LGTF). We clipped the sequencing adapters from the raw reads, which were subsequently denoised and clustered into Amplicon Sequence Variants (ASV) using dada2 (vers. 1.14)^54^ as implemented in QIIME2^55^. After taxonomic assignment, autotrophic community members were extracted from the 18 S dataset and merged with cyanobacterial reads from the 16 S dataset, accounting for the number of reads in both datasets. Multivariate and diversity analyses were performed in R using the packages vegan, betapart, metacom, and Venn Diagram.

### Confocal laser scanning microscopy

Biofilm samples (~5 mm × 5 mm) were embedded in optimal cutting temperature compound at the end of the experiment and frozen (−20 °C). Thin sections (50 μm) were cryosectioned and immediately imaged by confocal laser scanning microscopy (Leica SP8 FLIM equipped with a Supercontinuum White Light Laser). Chlorophyll *a* autofluorescence was excited at 670 nm and recorded between 685 and 750 nm. Phycocyanin autofluorescence was excited at 594 nm and collected between 640 and 660 nm.

### Numerical model

A computational model was built in COMSOL Multiphysics 5.5 based on the finite element method. It takes into consideration both the bulk flow (water) and the biofilm (water-saturated porous media) part in a two-dimensional domain. The model ensures mass conservation in 2D and thus flow over protruding biofilm structures such as tufts occurs entirely over their top, while in reality (3D) a part of the flow is diverted laterally around tufts. For a given streamwise velocity, our model may thus slightly overestimate pressure differences between the up- and downstream sides of a patch. The model solves for both fluid flow and O_2_ transport. Two different transport conditions were simulated, namely the consumption and production of O_2_ by the biofilm in dark and light conditions, respectively. The simulations were used to predict the following parameters: O_2_ production (NP)/consumption (R) rate [mol O_2_ m^−3^ s^−1^)], biofilm permeability K [m^2^] and porosity ε [−], effective diffusivity coefficient C = D_eff_/D_aq_ [−], turbulent Prandtl number P_rt_ = ν_t_/D_t_, with ν_t_ the eddy viscosity [m^2^ s^−1^], and the eddy diffusivity D_t_ [m^2^ s^−1^]. To obtain the parameter values an optimization procedure was carried out in COMSOL as described below.

The biofilm geometries in the model are representative of realistic biofilm patches reconstructed from OCT images (Supplementary Fig. 7). The domain had a constant height of 0.005 m, while its width varied from ~0.004 to ~0.01 m, according to patch type. The structure was meshed using an unstructured grid made of triangles and quads elements with a total number of ~5 × 10^4^ cells. The maximum and minimum element sizes were ~1 × 10^-4^ and ~1 × 10^−5^ m, respectively. The mesh size was refined at the fluid–biofilm interface and an inflation layer was created at the bottom of the biofilm to simulate boundary layer formation (Supplementary Fig. 8). This grid allowed a convergence with residuals lower than ~1 × 10^−6^.

For the boundary conditions of the flow problem, a velocity profile was set at the inlet, which was obtained from the fitting of LDV measurements with a quadratic polynomial, considering only the points within the domain height (0.005 m = 14 points). This profile likely over-predicts actual velocities within the porous biofilm, as seen by the faster velocities near the inlet compared to the rest of the domain (Fig. 3). However, an equilibrium is reached quickly, i.e., velocity in the porous medium is quite uniform ~1 mm away from the inlet (Fig. 3). At the outlet, a zero static pressure was assumed. A no-slip condition was set at the bottom of the biofilm and a slip condition was selected for the top of the flow domain to account for the remaining fluid above the considered domain.

Regarding the transport problem, individual concentration profiles for each biofilm type obtained from microsensor profiling were imposed as an inlet boundary condition, while at the outlet an outflow condition was imposed. A zero flux was set at the top and bottom of the domain, and a constant rate of production (or consumption) was set in the biofilm domain. The entire domain was initialized with zero velocity and zero O_2_. We include turbulence using a Reynolds-averaged model with constant eddy viscosity/diffusivity and assumed steady-state conditions of the mean flow. Prior to optimization^56^, an initial guess for the eddy viscosity was obtained by estimating the integral length scale of the turbulence (~7% of the water depth) and turbulent velocity fluctuations (u′ equal to friction velocity), and the turbulent Prandtl number was initialized as P_r_ = 1. Even though in reality the eddy viscosity decays toward the fluid–biofilm interface, this simple model is sufficient to reproduce the sharp transition to a flat concentration profile from the biofilm to the bulk liquid flow.

### Governing equations

The water flow at the top of the biofilm was modeled as steady, turbulent, and incompressible. Therefore, we applied the Reynolds-averaged Navier–Stokes (RANS) and continuity equations. The transport of O_2_ is also considered steady, since the comparison is done with experimental data collected at steady-state conditions. The Eq. (3) of transport is:

3 $$- D\nabla^2 {c} + {\overline{u}} \nabla {c} = R$$

where $\overline u$ is the flow velocity vector, *c* is the O_2_ concentration [mol m^−3^], D is the O_2_ diffusivity in water [m^2^ s^−1^] and *R* is the production (NP)/consumption rate of O_2_ [mol s^−1^]^56^. The ratio between advective and diffusive fluxes was calculated as in Eq. (4).

4 $$Jc/Jd = \left| {\overrightarrow u C} \right|/\left| { - D_{eff}\nabla C} \right|$$

A ratio larger than unity indicates the dominance of advective over diffusive fluxes, whereas a ratio smaller than one indicates the dominance of diffusive fluxes.

### Parameters optimization

To optimize the parameter listed in Table 2, the optimization module in COMSOL Multiphysics 5.5 was employed. For each patch type and each flow and light condition, one transect of O_2_ concentration profiles was used as Least-Squares Objective features to create an objective function of the sum of squared differences between experimental data and a corresponding expression calculated by the model. The model expression was evaluated using interpolation on the feature selection, at measurement locations. Then, the Nelder–Mead algorithm^57,58^ was used to optimize the parameters. The solver uses geometrical reflections, expansions, and contractions to improve the points in the simplex. The optimality tolerance was set to 1 × 10^−4^ for each controlled variable (parameter ranges used in the optimization procedure are reported in Supplementary Table 2). Given the effects of missing lateral flow diversion in our model (compared to a 3D scenario), the optimization of permeability may have yielded a lower permeability (to provide the same advective transport compatible with the experimental concentration profile within the biofilm but based on a lower pressure gradient). One might thus expect larger differences in permeability between KDP and the other biofilm types in 3D than in 2D.

Profiles modeled using the optimized parameters were validated against O_2_ concentration profile transects. The Pearson correlation coefficients between measured and predicted O_2_ concentrations were 0.4 ± 0.3 (mean ± standard deviation) under slow, and 0.6 ± 0.2 under fast flow (Supplementary Fig. 9). The quality of the fit between measured and predicted O_2_ concentrations was generally low for transects across very tall KDP under fast flow. The height of these structures exceeded the OCT imaging field, and the geometry used to model O_2_ dynamics was therefore inaccurate. In addition, profiles in the proximity of the boundary of the modeled domain presented a lower fit as a consequence of the velocity profile at the inflow boundary, which was measured without the biofilm. With increasing downstream distance, the velocity profile gradually adjusted to the given roughness conditions of the different biofilm transects, and generally the quality of the fits improved accordingly.

We calculated the average wall shear stress τ over the biofilm surface. The friction velocity is given by Eq. (5).

5 $$u^ \ast = \sqrt {\tau /\rho }$$

where ρ is the fluid density. The viscous length scale δ was estimated as in Eq. (6).

6 $$\delta = \frac{\nu }{{u^ \ast }}$$

where ν is the kinematic viscosity of water. Finally, the DBL scale was estimated using Eq. (7).

7 $${\mathrm{DBL}}_{{\mathrm{scale}}} = \delta /\sqrt {Sc}$$

where *Sc* = ν/*D*_w_ is the Schmidt number and *D*_w_ is the diffusion coefficient of oxygen in water. However, it should be noted that our numerical model realizations clearly depend on the chosen biofilm geometry and that parameter optimizations can be trapped in local maxima. We chose representative biofilm structures and provided plausible parameter ranges for optimizations, yet, this does not allow us to quantify uncertainties associated with these model predictions. The use of smoothed OCT images may thus reflect idealized biofilm structure and function; however, we deem this idealization well suited for upscaling. Consequently, volume-weighted metabolic rates were used for upscaling to the entire biofilm landscape. Given the absence of evidence for effects of metabolic activity extending across neighboring biofilm patches (e.g., Fig. 1f), the spatial arrangement of the different patch types and spatial interactions were not considered.

### Reporting Summary

Further information on research design is available in the Nature Research Reporting Summary linked to this article.

## Supplementary information

Supplementary Material

Reporting Summary

## Data Availability

Experimental data, as well as ASV tables, are available on Figshare (10.6084/m9.figshare.c.5687416.v1). Raw sequencing reads were deposited at the European Nucleotide Archive under accession number PRJEB48423.
