# Supplementary material for: Biophysical properties at patch scale shape the metabolism of biofilm landscapes
Source: NPJ Biofilms Microbiomes. 2022 Feb 3;8:5. doi: 10.1038/s41522-022-00269-0 (PMC8813951; doi:10.1038/s41522-022-00269-0)
Supplement: Supplementary file 1 — Supplementary Material [file 41522_2022_269_MOESM1_ESM.pdf]

Supplemental Information

Biophysical properties at patch scale shape the metabolism of biofilm landscapes

Anna Depetris, Giorgia Tagliavini, Hannes Peter, Michael Kühl, Markus Holzner, Tom Battin

Supplementary Table 1. Estimated porosity (mean ± standard deviation) from the OCT scans of biofilms growing under fast and slow flow, by patch type (CDB, DDP, and KDP). Porosity was estimated considering the surface layer of the biofilm (either 0.1 or 0.2 mm thick) and testing two gray-level thresholds (125 and 130) to separate biomass and voids.

Flow	Parameters	CDB	DDP	KDP
Fast	0.1 μm; gray-level :125	0.29 ± 0.22	0.26 ± 0.25	0.59 ± 0.22
	0.1 μm; gray-level :130	0.38 ± 0.24	0.34 ± 0.28	0.71 ± 0.21
	0.2 μm; gray-level :125	0.35 ± 0.16	0.35 ± 0.18	0.64 ± 0.16
	0.2 μm; gray-level :130	0.47 ± 0.16	0.46 ± 0.19	0.76 ± 0.15
Slow	0.1 μm; gray-level :125	0.29 ± 0.24	0.29 ± 0.25	0.53 ± 0.23
	0.1 μm; gray-level :130	0.39 ± 0.25	0.37 ± 0.26	0.64 ± 0.23
	0.2 μm; gray-level :125	0.36 ± 0.16	0.36 ± 0.17	0.59 ± 0.16
	0.2 μm; gray-level :130	0.47 ± 0.16	0.46 ± 0.17	0.71 ± 0.15

Supplementary Table 2. Features of the distributions in O_2 concentration measurements within the biofilm of different patch types (CDB, DD, and KDP).

Flow	Patch type	Light condition	Median [μmol l^{-1}]	max – min [μmol l^{-1}]
Slow	CDB	dark	307	332 – 229
		light	486	684 – 344
	DDP	dark	289	333 – 183
		light	555	723 – 341
	KDP	dark	304	333 – 226
		light	525	697 – 341
Fast	CDB	dark	276	329 – 219
		light	464	623 – 336
	DDP	dark	245	331 – 38
		light	515	682 – 338
	KDP	dark	316	328 – 212
		light	362	604 – 324

Supplementary Table 3. Parameter ranges used in the optimization procedure. CDB, DDP, and KDP indicate the different patch types. For the definition of each variable see numerical model section.

	DDP	CDB	KDP
Consumption rate [mol m^{-3} s^{-1}]	$-2e^{-3}$ — $-4e^{-4}$	$-2e^{-3}$ — $-4e^{-4}$	$-2e^{-3}$ — $-4e^{-4}$
Production rate [mol m^{-3} s^{-1}]	$1e^{-4}$ — $1e^{-2}$	$1e^{-4}$ — $1e^{-2}$	$1e^{-4}$ — $1e^{-2}$
O_2 diffusivity (D_{eff}/D_{aq})	0.5 — 0.7	0.5 — 0.7	0.7 — 1
Porosity	0.04 — 0.63	0.04 — 0.63	0.04 — 0.63
Permeability [m^2]	$1e^{-11}$ — $1e^{-8}$	$1e^{-11}$ — $1e^{-8}$	$1e^{-11}$ — $1e^{-8}$
Turbulent Prandtl number	0.5 — 1	0.5 — 1	0.5 — 1
Eddy diffusivity [m^2 s^{-1}]	$1e^{-5}$ — $1e^{-4}$	$1e^{-5}$ — $1e^{-4}$	$1e^{-5}$ — $1e^{-4}$

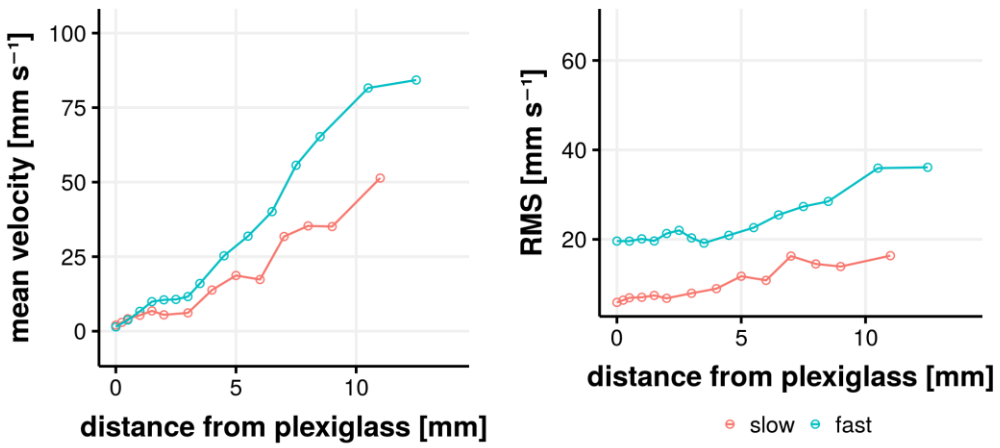

Supplementary Figure 1. Flow velocity and turbulent kinetic energy (measured as root mean square (RMS) velocity fluctuations) profiles measured by LDV at the slow- and fast-flow extremes of the velocity gradient.

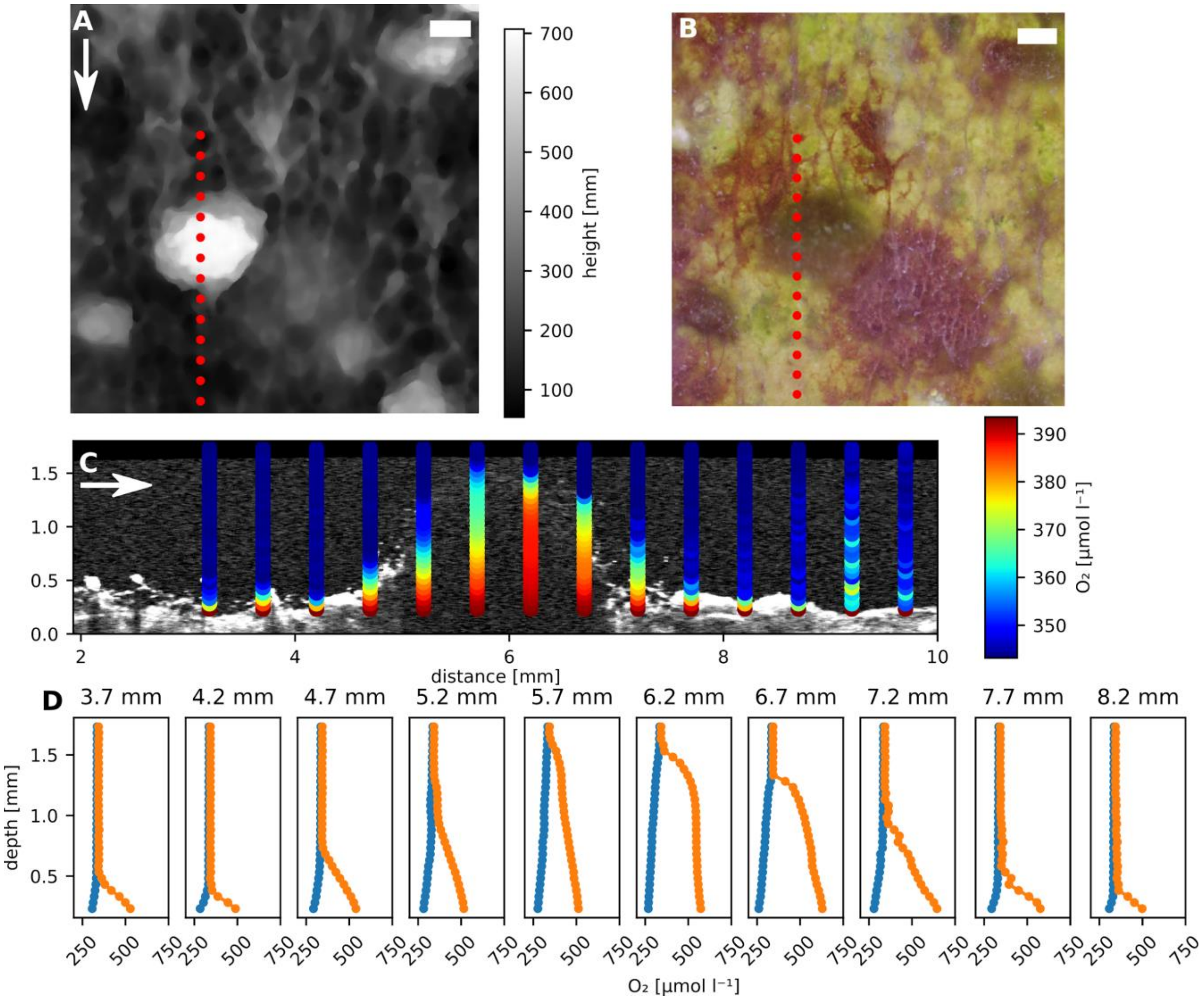

Supplementary Figure 2. Example of a transect of O_2 concentration profiles over a KDP under slow flow, imaged by OCT (A) and macro-photography (B). The locations of the profiles (spaced 0.5 mm) are indicated by the red dots. Scale-bars: 1 mm. The corresponding OCT B-scan is overlaid with the O_2 concentration measurement locations, color coded with the relative concentration in illuminated conditions (C). The same profiles under light (orange) and dark (blue) conditions are contrasted in panel D. Arrows indicate flow direction.

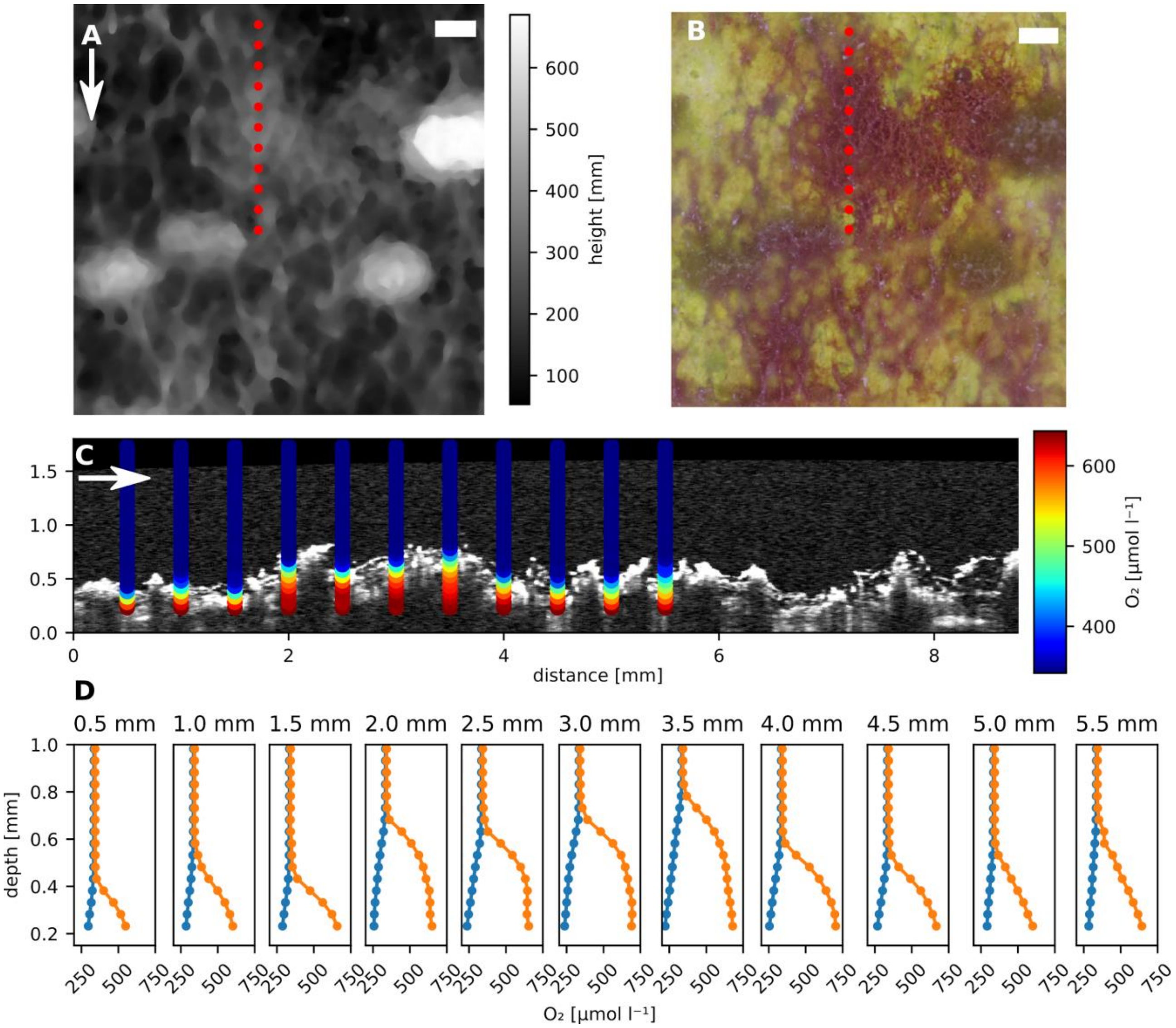

Supplementary Figure 3. Example of a transect of O_2 concentration profiles over a DDP under slow flow, imaged by OCT (A) and macro-photography (B). The locations of the profiles (spaced 0.5 mm) are indicated by the red dots. Scale-bars: 1 mm. The corresponding OCT B-scan is overlaid with the O_2 concentration measurement locations, color coded with the relative concentration in illuminated conditions (C), while the same profiles are plotted (D). Arrows indicate flow direction.

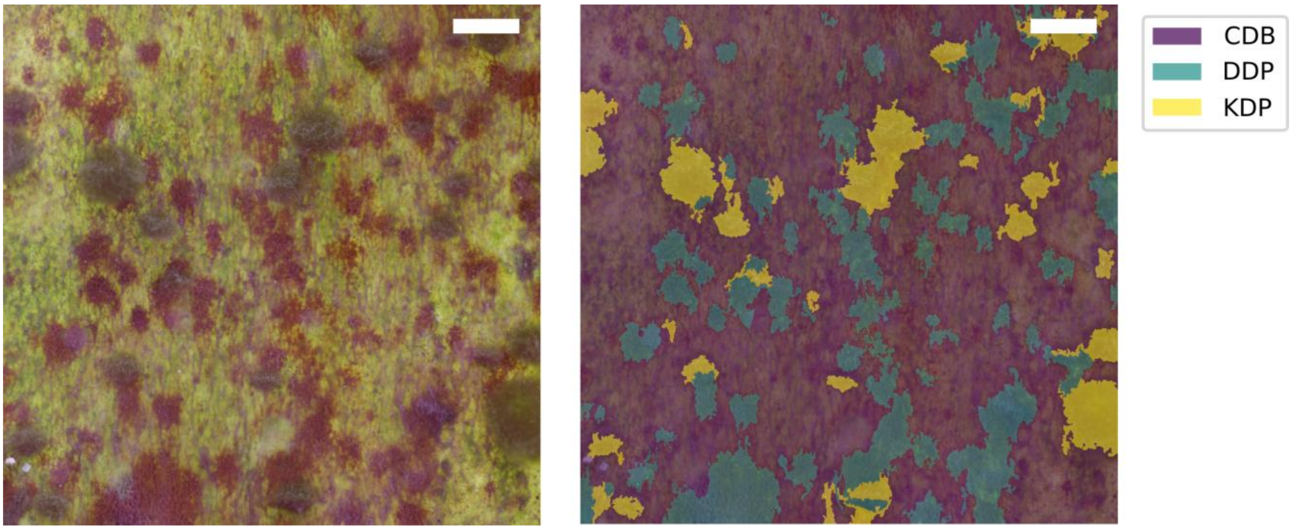

Supplementary Figure 4. Segmentation of the three patch types (CDB, DDP and KDP) from the macro-photograph (A), based on their color (B). Scale-bars: 5 mm.

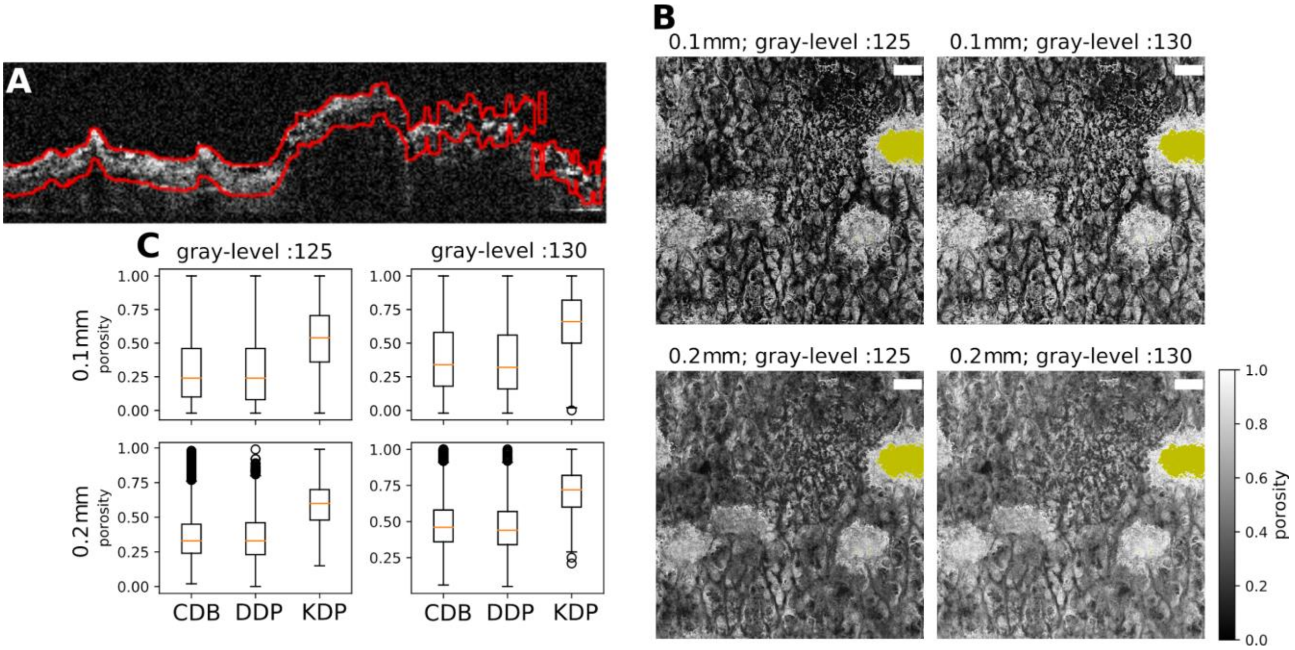

Supplementary Figure 5. Porosity of the biofilm was estimated from the OCT B-scans as the ratio of void volume over the total volume in the surface layer of the biofilm, indicated by the red lines (A, *Methods)*. Two layer widths (0.1 mm and 0.2 mm) and two gray-level thresholds (120, 130) were evaluated, and the biofilm portions that were too tall to be scanned by OCT were excluded (in yellow) (B). Based on the segmented macro-photographs (*Methods)*, the porosity distribution for structures of each patch type was computed (C).

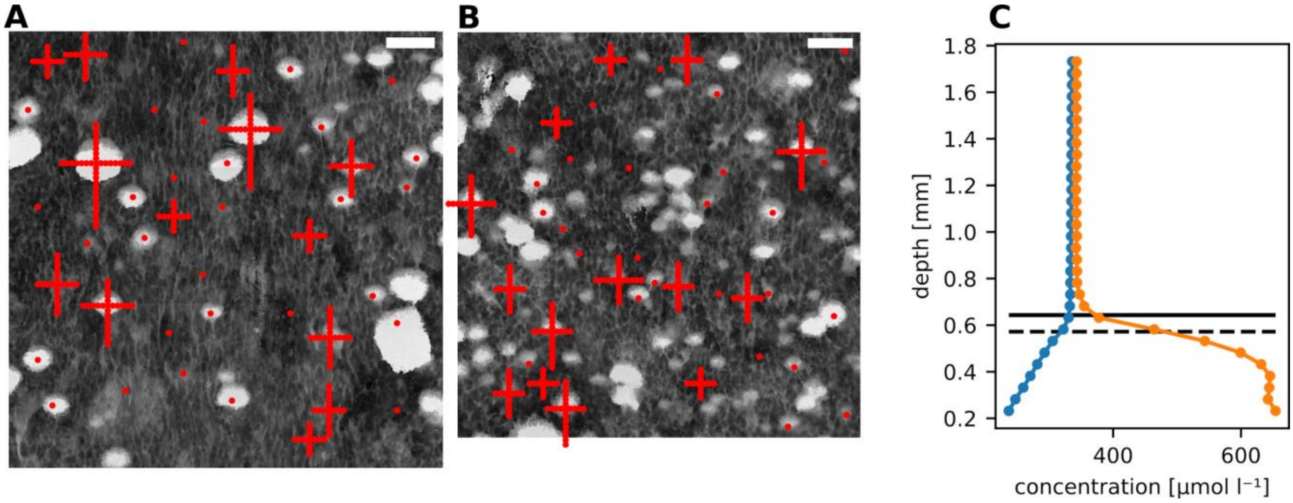

Supplementary Figure 6. Locations of each measured O_2 concentration profile (red dots) with respect to the OCT-derived DEMs under fast and slow flow are shown (A and B, respectively). The profiles in transects (flow- and span- directions) are spaced 0.5 mm. Scale bars: 5 mm. For each location, O_2 concentration profiles were measured in both light and dark conditions. An example of a profile measured on CDB under fast flow in dark (blue) and light (orange) is shown (C). The dashed line shows the position of the biofilm surface estimated from the profile inflection point, the full line indicates the position of the biofilm surface estimated from the OCT scan.

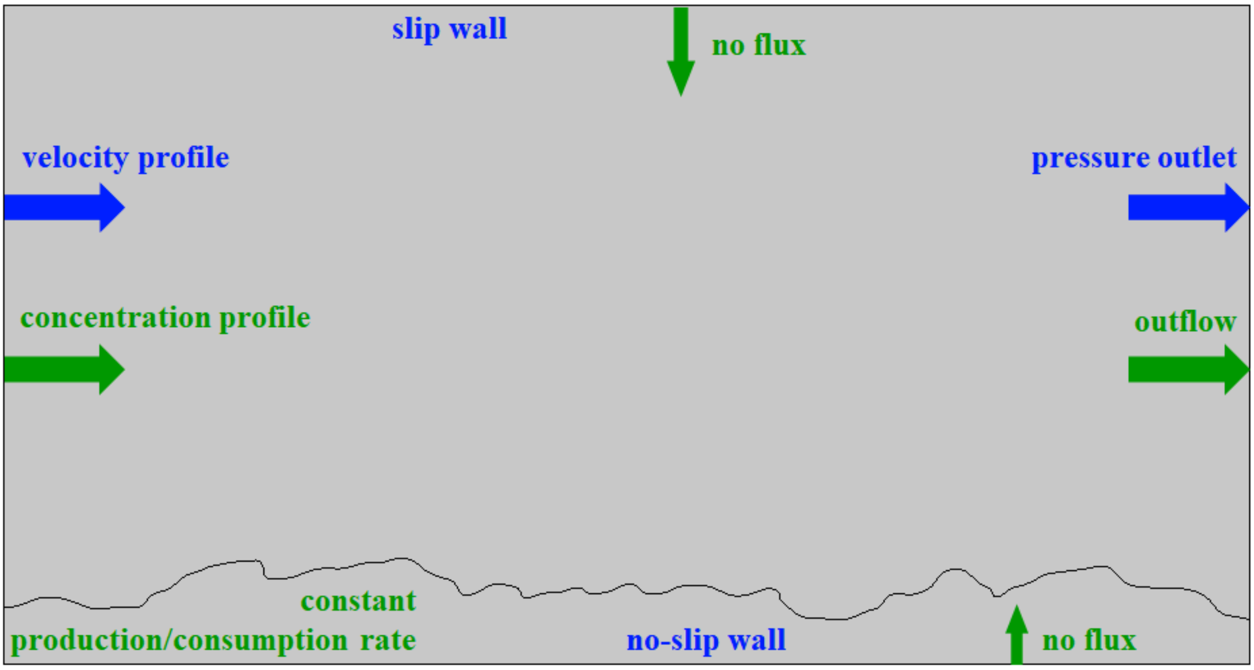

Supplementary Figure 7. Example of domain geometry (DDP) and boundary conditions of the computational model.

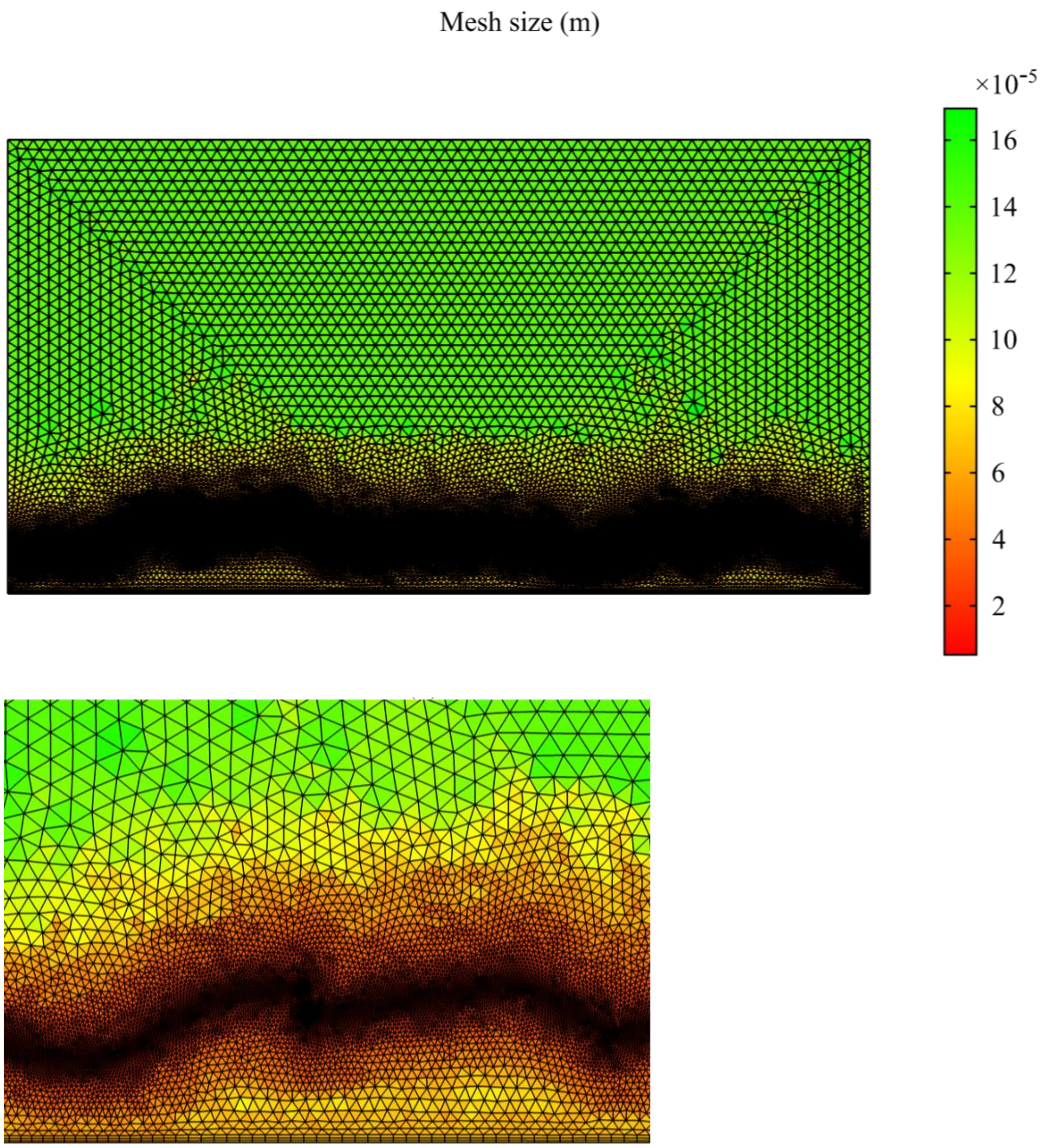

Supplementary Figure 8. Computational domain grid size in [m] (a), with the detail (b) of the biofilm discretization, in which both the refinement at the fluid-biofilm interface and the inflation layer are visible.

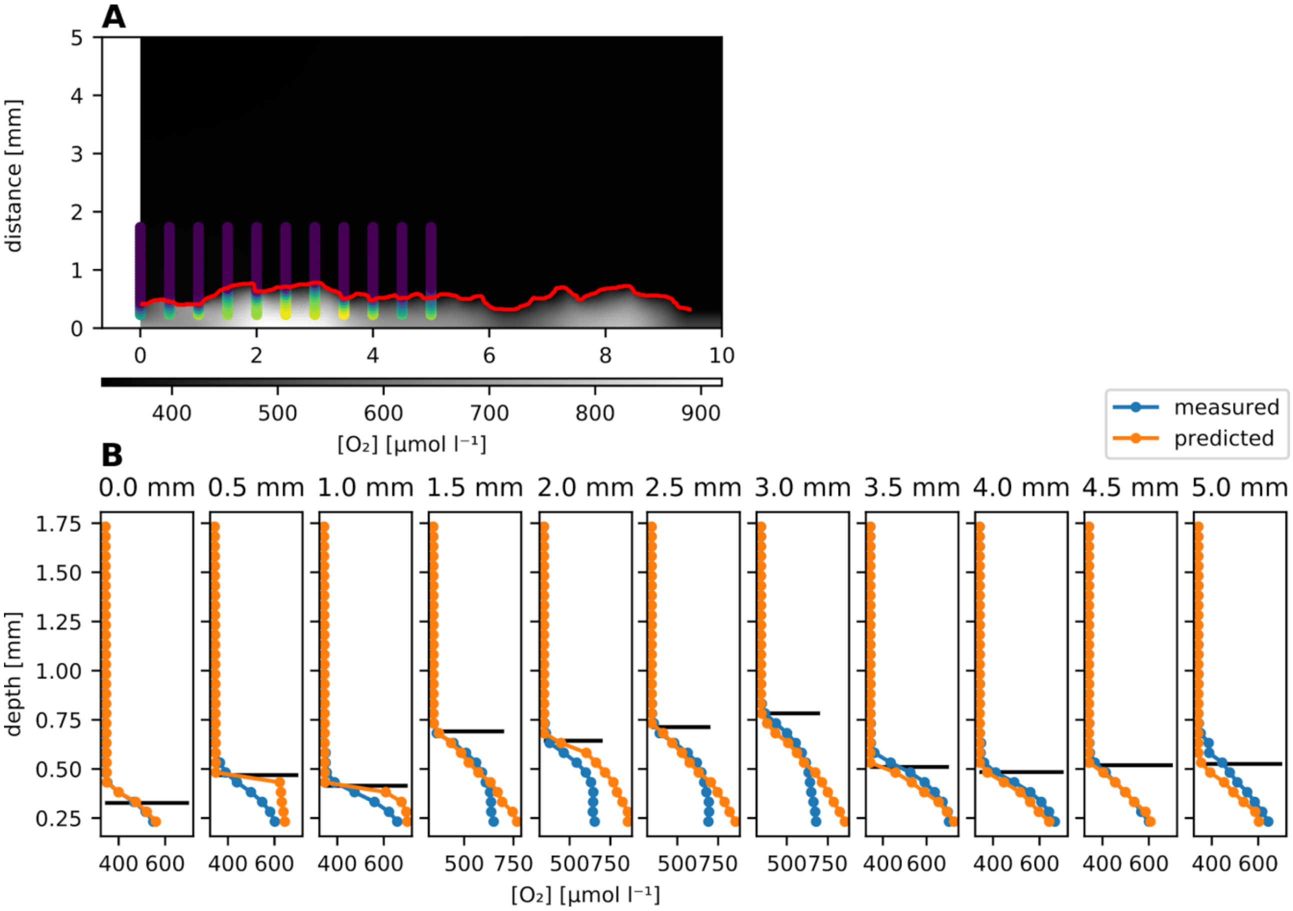

Supplementary Figure 9. Comparison between measured and predicted O_2 concentration profiles along a transect. The numerically modeled O_2 concentration field around an illuminated DDP under slow flow is shown (A). The surface of the biofilm, scanned by OCT, is indicated by the red line. The points indicate the locations in which the O_2 concentration was measured with a microsensor, color-coded based on the respective concentrations. The model was parametrized by optimizing the fit between predicted and measured O_2 concentrations (B). Note the divergence of predicted and measured O_2 concentration profiles in deeper layers of thick biofilm portions.
