# Supplementary material for: Biophysical properties at patch scale shape the metabolism of biofilm landscapes
Source: NPJ Biofilms Microbiomes. 2022 Feb 3;8:5. doi: 10.1038/s41522-022-00269-0 (PMC8813951; doi:10.1038/s41522-022-00269-0)
Supplement: Supplementary file 2 — Reporting Summary [file 41522_2022_269_MOESM2_ESM.pdf]

nature portfolio

Corresponding author(s): Hannes Peter

Last updated by author(s): Nov 18, 2021

Reporting Summary

Nature Portfolio wishes to improve the reproducibility of the work that we publish. This form provides structure for consistency and transparency in reporting. For further information on Nature Portfolio policies, see our Editorial Policies and the Editorial Policy Checklist.

Statistics

For all statistical analyses, confirm that the following items are present in the figure legend, table legend, main text, or Methods section.

n/a	Confirmed	
☐	☒	The exact sample size (n) for each experimental group/condition, given as a discrete number and unit of measurement
☐	☒	A statement on whether measurements were taken from distinct samples or whether the same sample was measured repeatedly
☐	☒	The statistical test(s) used AND whether they are one- or two-sided<br>*Only common tests should be described solely by name; describe more complex techniques in the Methods section.*
☒	☐	A description of all covariates tested
☐	☒	A description of any assumptions or corrections, such as tests of normality and adjustment for multiple comparisons
☐	☒	A full description of the statistical parameters including central tendency (e.g. means) or other basic estimates (e.g. regression coefficient) AND variation (e.g. standard deviation) or associated estimates of uncertainty (e.g. confidence intervals)
☒	☐	For null hypothesis testing, the test statistic (e.g. F, t, r) with confidence intervals, effect sizes, degrees of freedom and P value noted<br>*Give P values as exact values whenever suitable.*
☒	☐	For Bayesian analysis, information on the choice of priors and Markov chain Monte Carlo settings
☒	☐	For hierarchical and complex designs, identification of the appropriate level for tests and full reporting of outcomes
☒	☐	Estimates of effect sizes (e.g. Cohen's d, Pearson's r), indicating how they were calculated

Our web collection on statistics for biologists contains articles on many of the points above.

Software and code

Policy information about availability of computer code

Data collection	OCT data was collected using previously published pipeline. A reference is provided. Oxygen microprofiles were recorded using a commercial software, a reference is provided.
Data analysis	Numerical simulations and statistical analyses were performed using R, python and Matlab. References to algorithms and packages are provided.

For manuscripts utilizing custom algorithms or software that are central to the research but not yet described in published literature, software must be made available to editors and reviewers. We strongly encourage code deposition in a community repository (e.g. GitHub). See the Nature Portfolio guidelines for submitting code & software for further information.

Data

Policy information about availability of data

All manuscripts must include a data availability statement. This statement should provide the following information, where applicable:

- Accession codes, unique identifiers, or web links for publicly available datasets
- A description of any restrictions on data availability
- For clinical datasets or third party data, please ensure that the statement adheres to our policy

Experimental data as well as ASV tables are available on Figshare (https://doi.org/10.6084/m9.figshare.c.5687416.v1). Raw sequencing reads were deposited at the European Nucleotide Archive under accession number PRJEB48423.

Field-specific reporting

Please select the one below that is the best fit for your research. If you are not sure, read the appropriate sections before making your selection.

☐ Life sciences ☐ Behavioural & social sciences ☒ Ecological, evolutionary & environmental sciences

For a reference copy of the document with all sections, see nature.com/documents/nr-reporting-summary-flat.pdf

Ecological, evolutionary & environmental sciences study design

All studies must disclose on these points even when the disclosure is negative.

Study description	A phototrophic biofilm was grown in a flume and the physical structure was recorded using OCT and macrophotography. Amplicon sequencing was used to probe the composition and diversity of three different patch types. Oxygen microprofiling was used to describe the distribution of oxygen within biofilm patches. Numerical simulations were used to derive process rates, which were used to upscale from the patch to the biofilm landscape level.
Research sample	A multi-species phototrophic biofilm was used in the experiment. These are relevant for the ecology of streams and rivers.
Sampling strategy	Samples were taken at the extremes of a hydraulic gradient. OCT imaging covered the entire area. Triplicated samples for sequencing were collected. Numerous (hundreds) of oxygen profiles were measured.
Data collection	OCT data was collected using an automated (robotic) device, as previously desribed (referenc provided). Samples for sequencing were prepared by laboratory assistants; sequencing was performed by a specialized Sequencing platform (name provided in manuscript). Oxygen microprofiles were semi-automatically acquired, using the same robotic positioning device. Calibrations were performed to assure stability of the measurements.
Timing and spatial scale	Biofilm was grown for 30 days. Samples (destructive) were taken during a single day.
Data exclusions	Non-phototrophic 18S rRNA gene sequences (based on taxonomic classification) were excluded. We deem the small-scale sampling insufficient to describe eukaryotic communities other than the main phototrophic community members. Some oxygen profiles were excluded, mainly because of insufficient sampling depth (within the biofilm).
Reproducibility	Growing a multi-species biofilm from a natural source (e.g. lake or stream water) will always result in different communities. This can not be replicated. However, we performed numerous comparable flume experiments and found that patchiness was common. This is supported by the literature. Hence, we argue while it will not be possible to regrow the exact same communities, the internal oxygen distributions are representative for patch types typically found in nature.
Randomization	No randomization was required.
Blinding	Blinding was not required.

Did the study involve field work? ☐ Yes ☒ No

Reporting for specific materials, systems and methods

We require information from authors about some types of materials, experimental systems and methods used in many studies. Here, indicate whether each material, system or method listed is relevant to your study. If you are not sure if a list item applies to your research, read the appropriate section before selecting a response.

Materials & experimental systems

n/a	Involved in the study
☒	☐ Antibodies
☒	☐ Eukaryotic cell lines
☒	☐ Palaeontology and archaeology
☒	☐ Animals and other organisms
☒	☐ Human research participants
☒	☐ Clinical data
☒	☐ Dual use research of concern

Methods

n/a	Involved in the study
☒	☐ ChIP-seq
☒	☐ Flow cytometry
☒	☐ MRI-based neuroimaging
